# Cardiac protection of germinated brown rice extract in rabbit model of chronic myocardial infarction

**DOI:** 10.1093/tas/txaa067

**Published:** 2020-05-20

**Authors:** Soontaree Petchdee, Wanpen Laosripaiboon, Nongpanga Jarussophon

**Affiliations:** 1 Department of Large Animal and Wildlife Clinical Sciences, Faculty of Veterinary Medicine, Kasetsart University, Kamphaeng Saen, Thailand; 2 Department of Chemistry, Faculty of Liberal Arts and Science, Kasetsart University, Kamphaeng Saen, Thailand

**Keywords:** cardioprotection, coronary heart disease, germinated brown rice, rabbit model

## Abstract

Ischemic heart disease is a leading cause of mortality in the world. This study aimed to investigate the cardioprotective effects of germinated brown rice (GBR) on a rabbit model of chronic myocardial infarction. Eighteen New Zealand white rabbits were randomly divided into three groups receiving: 1) regular rabbit food; 2) regular rabbit food plus vehicle; and 3) regular rabbit food plus GBR for 120 d. The left circumflex coronary artery was ligated to induce myocardial ischemia 60 d after starting the experiment (baseline). Heart functions were monitored by electrocardiography and echocardiography at 0, 30, and 60 d after coronary artery ligation. The incidences of heart rate (HR) and ventricular arrhythmias have been compared between groups. GBR showed the effects to prevent life-threatening ventricular tachycardia and electrocardiographic signs of myocardial ischemia in a model of arrhythmias. GBR consumption group exhibited significantly improved cardiac function and reduced the HR, along with reduced mean arterial pressure and plasma glucose level. The results demonstrated that GBR exerts cardioprotective effects against chronic myocardial injury in rabbits. These biological actions of GBR may explain the benefits gained from the use of GBR products as a possible prophylactic lifestyle intervention.

## INTRODUCTION

Coronary heart diseases cause a large number of deaths worldwide, and ventricular arrhythmia is a leading contributor to mortality from heart disease (Long III et al., 2015). It is well established that hypertension, hyperglycemia, dyslipidemia, and sedentary lifestyle are the important risk factors for coronary heart disease (Fakhrzadeh et al., 2016; Ladapo et al., 2016). In recent years, alternative health care using diet and dietary components has become increasingly popular due to its beneficial effects discovered (Elsas et al., 2010; Najafabad and Jamei, 2014). In particular, the protective effects in metabolic diseases have been attributed to the consumption of wholegrain foodstuff, especially germinated brown rice (GBR; Ling et al., 2001, 2002; Lillioja et al., 2013). Germination is a natural process during the growth period of seeds; the germination process improves texture and increases the useful compounds of the grain (Usuki et al., 2008; Imam et al., 2013). Germinated brown rice is a biotransformation product of brown rice; it is known that GBR showed excellent benefits on health promotion and disease prevention (Ho et al., 2012).

In contrast to the extensive studies on the protective effects of GBR on central nervous system diseases, fewer studies on the health effect of GBR on coronary heart diseases have been published. According to several studies, GBR is a good source of phytochemicals with various pharmacological activities, such as antiatherosclerotic, antidepressant, anti-inflammatory, antidiabetic, and hypolipidemic (Usuki et al., 2007; Shen et al., 2016). However, the effects of GBR on cardiac function have rarely been reported. In this study, the cardioprotective effects of GBR have been investigated in a chronic myocardial infarction (MI) model in rabbits.

## MATERIALS AND METHODS

### Preparation of Germinated Brown Jasmine Rice

Brown jasmine rice (*Oryza sativa* L.) was obtained from Sakonnakorn province, Thailand, in 2014. Washed grains (100 g) of brown jasmine rice were soaked in water at 35 °C for 12 h. After that, the seeds were placed onto moist filter papers and covered for 24 h of germination. After germination, the water was drained off, and grains were rewashed. These grains were then taken out. The germinated seeds were finely ground before analysis and preparation of the germinated brown and red jasmine rice extract powder. The samples were stored at −20 °C until used.

### Determination of Bioactive Composition

#### Determination of total phenolic content

Total phenolic content (TPC) was determined by the Folin–Ciocalteu method (Chen et al., 2016). Briefly, the extracts (free and bound phenolic compounds) were introduced into test tubes, and then 50% Folin–Ciocalteau reagent were added. The reaction was treated with 2% Na2CO3. The mixture was incubated at room temperature for 30 min. The absorbance was measured at 750 nm using a UV-Vis spectrophotometer. Gallic acid was used as a chemical standard of calibration. The TPC data was expressed as milligram gallic acid equivalents (GAE) per 100 g of dry matter (mg GAE/100 g).

#### Determination of total flavonoid content

Total flavonoid content (TFC) was determined using colorimetric method, the germinated ground rice sample was extracted with 80% ethanol for 2 h. Each extract was centrifuged at 4,000 rpm for 20 min. A 0.3-mL aliquot of the above extract was added to a tube containing 1.5 mL of distilled water. The extract was then mixed with 0.09 mL 5% NaNO_2_ solution, and the mixture was left at room temperature for 5 min; 0.18 mL 10% AlCl_3_·6H_2_O solution was added, and the mixture solution was left for 10 min; 0.6 mL of 1 M NaOH solution was then added to the reaction. Distilled water was added to make the total volume 3 mL. The absorbance was determined at 510 nm using a Shimadzu UV-1800 spectrometer. Total flavonoid contents were expressed as milligram catechin equivalents (CE) per 100 g DW of sample. Distilled water was added if the absorbance measured was over the linear range of the catechin standard curve.

#### Determination of γ-aminobutyric acid

γ-Aminobutyric acid (GABA) contents in germinated rice were determined by using Zhang et.al. (2014) method. One gram of germinated rice of each sample was weighed into a plastic tube and 5 mL deionized water was added. The mixture was oscillated and extracted for 1 h, then filtered. Half a milliliter of the filtrate was collected to which was added 0.2 mL of 0.2 mol/L borate buffer (pH 9.0), 1 mL of 6% phenol, and 0.4 mL of 10% sodium hypochlorite. After intensive oscillation, the mixture was put in boiling water for 10 min, and then put in an ice bath for 20 min, and continually oscillated until a blue color appeared. Finally, 2 mL of 60% ethanol were added to the mixture, and the sample was analyzed colorimetrically at 630 nm wavelength.

#### Determination of phytic acid

The method to determine the phytic acid content in germinated rice was modified from Fable et al. (2002) method. HCl–Na_2_SO_4_ solution (50:50 v/v) was added to 1 g dried sample and left for 90 min with intermittent agitation. Ten milliliter of the transparent floating liquid of the previous extraction (filtered), 10 mL of Fe (III) solution, and 20 mL of sulfosalicylic acid solution are placed in a neutral glass tube and heated for 15 min in a boiling water bath. The clean, floating material was separated and placed in a 250-mL precipitation beaker, filling it up to around 200 mL with deionized water and increasing pH to 2.5 ± 0.5. The solution was titrated until bright yellow appeared. The percentage of phytic acid in sample is deduced from the following equation:

$$\begin{array}{l} Phytic\;acid\;\left(\%\right)\;=\;1.32\left(10-V\right)/P \end{array}$$

where V = Ethylenediamine Tetraacetic Acid solution volume (milliliters), P = sample weight (grams).

### Antioxidant Assay for 2,2-Diphenyl-1-Picrylhydrazyl Radical Scavenging Activity

The free radical scavenging activity plant extract was evaluated using the stable radical 2,2-diphenyl-1-picrylhydrazyl (DPPH). A series of difference concentration, 5, 10, and 25 mg/L were prepared. Then 1.5 mL of each diluted plant extract was mixed with 1.5 mL of 0.20 mM DPPH in methanol. The mixture of difference extract concentrations and DPPH were placed in dark at 37 °C for 30 min. After incubating, the absorbance was measured at the wave length of 517 nm. The DPPH radical scavenging activity (%) was calculated by using the following formula:

$$\begin{array}{ll} Scavenging\;ef\;fect\;\left(\%\right)\;= & \;\left(1-A_{sample}/A_{control}\right)\\ & \;\times \;100\% \end{array}$$

### Bioaccessibilty of GBR

Bioaccessibility in the GBR extract was carried out by a three-step in vitro digestion, which includes oral, gastric, and intestinal absorption (Minekus et al., 2014). For the oral step, GBR extract (2 g) was mixed with simulated saliva fluid solution and α-amylase solution. Then 0.3 M CaCl_2_ was added and maintained at 37 °C for 30 min in a shaking water bath. For the gastric step, 0.5 mL of pepsin solution (11,000 U/mL) was added to the sample and maintained at pH 3 (0.1 M HCl) for 2 h in a shaking water bath at 37 °C. Then the sample was adjusted to pH 5 (1 M NaHCO_3_) and the further step was performed. The intestinal step was followed by the addition of 2.5 mL of pancreatin-bile solution. Then 40 μL of 0.3 M CaCl_2_ was added, and the pH was adjusted to 7.0 and incubated for 2 h in a shaking water bath at 37 °C. The samples were cooled in ice for 10 min and centrifuged at 4,600 rpm for 40 min at 4 °C. The supernatants were separated and analyzed for bioaccessibility by the HPLC system.

### Animals

New Zealand white rabbits weighing 2.5–3.0 kg were used in this study. Rabbits were randomly divided into three groups. The left circumflex coronary artery was ligated to induce chronic MI.

Group 1 (sham-operated group; *n* = 6): In this group, rabbits were subjected to the sham operation and were given regular rabbit food.

Group 2 (Control group; *n* = 6): MI was induced and rabbits were given a regular rabbit food.

Group 3 (GBR group; *n* = 6): MI was induced and rabbits were given regular rabbit food plus GBR as a supplement for 120 d. The nutrient compositions between regular rabbit food and rabbit food plus GBR supplement are shown in Table 1.

**Table 1.** The amount of nutrient compositions between regular rabbit food and rabbit food plus GBR supplement

	Regular rabbit food, %	Rabbit food with GBR supplement, %
Crude protein	12	12
Crude fat	2.5	2.5
Crude fiber	18	18
Calcium	0.3	0.3
Phosphorus	0.3	0.3
Salt	0.25	0.25
Vitamin A, IU/lb	2,500	2,500
Phenolic	—	0.3
Flavanoid	—	0.1
GABA	—	0.02
Phytic acid	—	0.01

### Myocardial Scar Generation (Chronic MI)

The surgical procedure to produce MI was performed according to a well-accepted technique with modifications (Ahn et al., 2004). Briefly, rabbits were anesthetized with pentobarbital sodium (45 mg/kg) and underwent a tracheal intubation for subsequent mechanical ventilation. The heart was exteriorized via left thoracotomy and subjected to left circumflex coronary artery occlusion with a 6-0 polypropylene suture. The heart was then quickly returned to its original location, the thorax was immediately closed, and the air was removed. Eight weeks after coronary occlusion, the rabbit were euthanized by an overdose of pentobarbital. The left ventricle was isolated and cut into slices. The slices were stained for 30 min at 37 ºC in a 0.1% solution of triphenyltetrazolium chloride (TTC). Myocardial infarction mass was expressed as a fraction of the total left ventricular (LV) weight.

### Measurement of Blood Pressure and Heart Function

**Figure 1.**
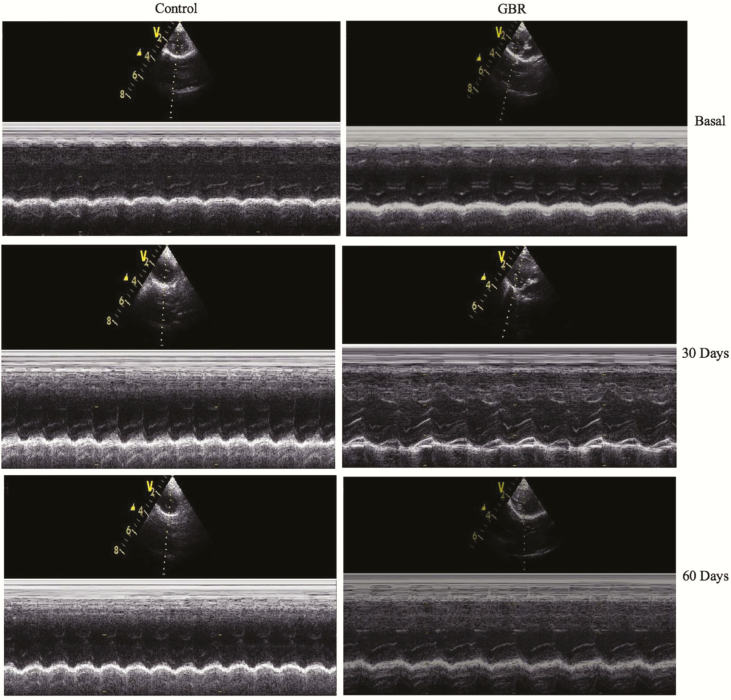
M-mode echocardiographic images of rabbit in control group (Control) and rabbit with GBR as a supplement (GBR) at base, 30 d, and 60 d after coronary artery ligation. Percentage of FS decreased after myocardial injury in the control group, while FS improved in the GBR supplement group.

Rabbits were sedated with propofol (AstraZeneca, London, UK) and placed on ventilator. Mean arterial pressure was measured by introduced transducer catheter (iWorx System, ADInstruments, New Zealand) into the femoral artery. Echocardiography was performed in right parasternal long axis view using 10 MHz transducer ultrasonic probe (Vivid 5s, GE) to assess the effects of GBR on LV contraction and transthoracic echocardiography was used to monitor LV fraction shortening (LVFS) as shown in Fig. 1 and Table 6. (Petchdee, 2019).

**Table 6.** Echocardiography parameters (Petchdee, 2019)

Echocardiography	Control (*n* = 6)	GBR (*n* = 6)	*P*
LVIDs, cm	0.73 ± 0.12	0.9 ± 0.11	0.33
LVIDd, cm	0.97 ± 0.19	1.2 ± 0.06	0.28
FS, %	23.67 ± 2.40*	32.0 ± 1.15*	0.01

Values reported as mean ± SEM.

### Electrocardiography Recording

A three-lead electrocardiography was performed throughout the coronary artery ligation. Oxygen saturation from pulse oximetry (SpO_2_), heart rate (HR), blood pressure (BP), S–T segment deviations, and arrhythmias were recorded throughout the procedure.

### Determination of Myocardial Infarct Size

After perfusion with TTC (Sigma), the heart was existed and the LV was transversely cut into 1.0-mm thick slices, which were incubated in TTC solution. The slices were fixed in 10% formalin overnight. Then they were placed between two transparent glasses and captured using a scanner at 600 dpi resolution. Images were analyzed using a scanner at 600 dpi resolution. The images were analyzed using image J software. The slices were then weighed for the determination of LV weight (Riess et al., 2009).

### Statistical Analysis

All data are presented as mean ± standard error of the mean. Results were analyzed by student’s paired *t*-test. A *P*-value <0.05 was considered statistically significant.

## RESULTS

The result of analysis of TPC, TFC, and antioxidant activity of GBR have been shown in Table 2. For animals weighing 2.5–3 kg, no significant difference in body weight and blood chemical profiles was observed in control and GBR groups on day 120 of GBR consumption. (Table 3). Results of echocardiography were shown in Fig. 1. The LVFS, diastolic left ventricular internal diameter (LVIDd), and systolic left ventricular internal diameter (LVIDs) were not different between control and GBR groups. However, the fractional shortening (FS) decreased significantly (*P* < 0.05) in the control group, while the FS increased in the GBR group. GBR might serve as a dietary supplement to improve contraction function on the ischemic heart disease.

**Table 2.** Classification and typical amounts of total phenolic, flavonoid, GABA, and phytic acid concentration present in GBR 100 g

Total phenolic, mg GAE/100 g DW	Total flavonoid, mg CE/g sample [DW]^2^	GABA, mg GABA/100g DW	Phytic acid, mg/g DW	Antioxidant activity EC_50_, μg/mL
150.18 ± 1.22	49.46 ± 3.39	13.00 ± 0.35	7.28 ± 0.40	81.57 ± 0.43

DW, dry weight.

**Table 3.** Hemodynamic and biochemical characteristics of rabbit model of myocardial injury administered with GBR and vehicle (Control) for 120 d

	Control (*n* = 6)	GBR (*n* = 6)	*P*
Blood work			
Hematocrit, %	41.63 ± 2.02	38.55 ± 2.26	0.33
Hemoglobin, g/dL	13.75 ± 0.93	12.7 ± 1.14	0.49
AST, U/L	67.33 ± 35.21	34.66 ± 18.51	0.43
ALT, U/L	75.76 ± 37.35	42.33 ± 16.23	0.43
BUN, mg%	25.7 ± 9.13	22.9 ± 4.63	0.79
Creatinine, mg%	1.62 ± 0.32	1.38 ± 0.30	0.59
Triglyceride, mmol/L	0.62 ± 1.57	0.59 ± 1.82	0.99
HDL, mmol/L	0.67 ± 0.14	0.65 ± 0.75	0.97
LDL, mmol/L	0.51 ± 0.35	0.49 ± 0.23	0.96

Values reported as mean ± SEM.

*P < 0.05 vs. control.

**Figure 2.**
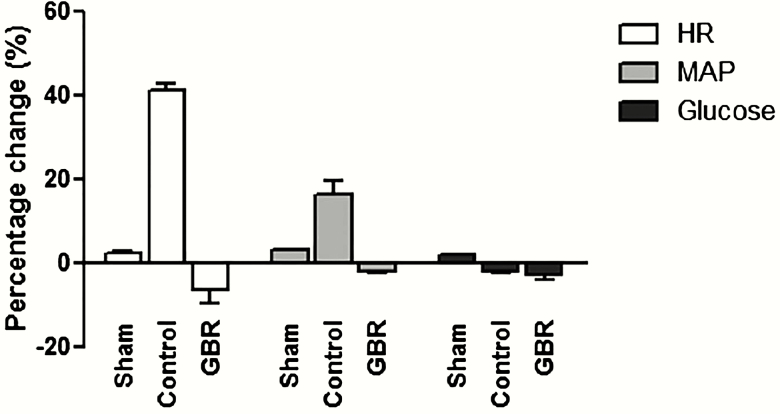
Cardiac protection effects of GBR on rabbit myocardial infarction model (*n* = 6). Graph showed percentage change on HR, MAP, and blood glucose level (Glucose) between days 0 and 60 after coronary artery ligation. The HR, BP, and plasma glucose levels were reduced in animals that were given GBR.

Intake of GBR as a supplement showed a trend to reduce the risk of cardiovascular diseases, such as hyperglycemia and hypertension. The HR was significantly lower than the basal value (day 0). On the other hand, in the MI (Control) group, HR was significantly higher than the basal HR (Fig. 2). There was no significant difference between the groups for mean arterial pressure (MAP) and plasma glucose level (Fig. 2).

The ventricular arrhythmias incidence was 67% (4/6) in the control group and 33% (2/6) in the GBR group. The alternans incidence was 67% (4/6) in the control group and 33% in the GBR group. Ventricular arrhythmia, S–T segment shift, and T-wave alternans showed a trend to reduce by the GBR consumption group (Table 4).

**Table 4.** Summary of arrhythmic events

		During coronary artery ligation		
	*n*	Alternans, %	VT, %	VF, %
Sham-operated	6	0	0	0
Control	6	67	33	33
GBR	6	33	33	0

VF, ventricular fibrillation; VT, ventricular tachycardia.

**Figure 3.**
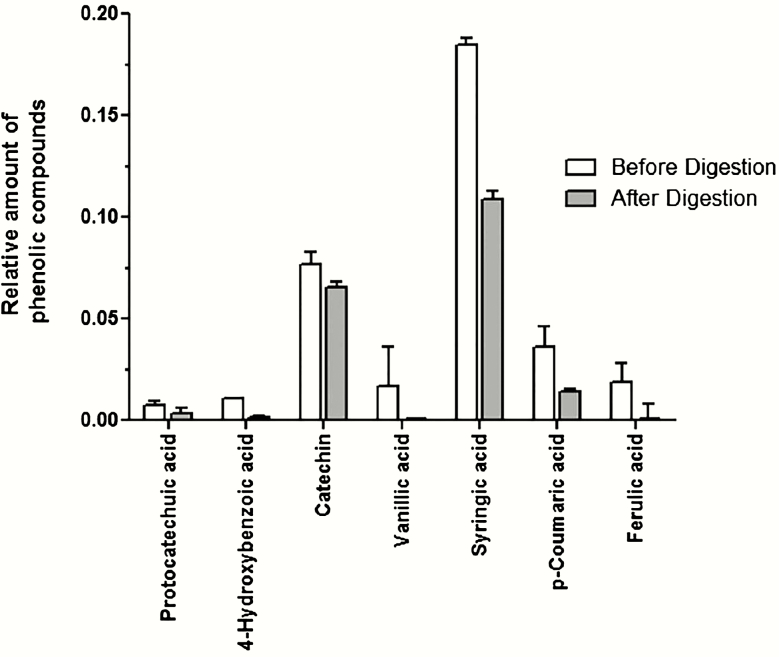
Phenolic profiles obtained before and after in vitro digestion. Values expressed as milligram of each compound per gram of sample. There was a reduction in the levels of phenolic compounds after in vitro digestion.

Figure 3 shows the phenolic profile obtained before and after in vitro digestion of GBR. Syringic acid was the major compound detected and qualified, followed by catechin, p-coumaric acid, and ferulic acid. As can be seen from the results in Fig. 3, there was a reduction in the levels of phenolic compounds after in vitro digestion. Our results showed that the phenolic compounds of GBR are affected by the in vitro digestion process. These changes can modify the bioavailability and further cardioprotection activity of GBR. However, further studies are needed to clarify these sequential events. As shown in Table 5, analysis of LV wall sections from control and GBR animals showed a significant reduction of infarct size in GBR group.

**Table 5.** Effect of GBR on myocardial infarct size after left circumflex coronary artery occlusion

	Total area, cm^2^	Infarct size area, cm^2^	Ratio
Sham-operated	3.18 ± 0.18	0	0
Control	4.06 ± 0.33	0.14 ± 0.01	31.24 ± 4.54
GBR	3.19 ± 0.11	0.18 ± 0.01	18.34 ± 0.68*

*p<0.05 vs. control

## DISCUSSION

We investigated the cardioprotection effects of GBR on heart function. In the present study, GBR consumption in rabbit model of chronic MI showed a considerable decrease in HR and improvement in heart functions, such as LVFS. Chronic intake for 16 wk of GBR also reduced the frequency of ventricular arrhythmias, including T-wave alternans, ventricular tachycardia, and ventricular fibrillation in the rabbit model of myocardial ischemia. The results of our study showed not only the incidence of ventricular arrhythmias but also the risk of atherosclerosis, such as hyperglycemia and hyperlipidemia. Plasma glucose level and cholesterol level were reduced in animals that were given GBR. These finding correlated with the results of some previous studies (Hsu et al., 2008).

The previous study demonstrated that the measurement of infarct size is a good predictor of sudden cardiac death. Myocardial infarction ≥23% of the LV volume was associated with major adverse cardiac events (Bulluck et al., 2016). The reduction of infarct size was also observed after the consumption of GBR. Then it may be possible that adding GBR as a supplement can help to prevent a sudden cardiac death. The cardiovascular disease prevention of GBR appears to be relative to direct effects on the biological properties, especially related to the antioxidant activity of the components from GBR. The active principle compounds of GBR includes phenolic contents, total flavonoid, and GABA. GBR contained the highest phenolic contents among the three main contents. In this present study, we only investigated the amount of phenolic content on the in vitro digestion experiment. Many unresolved questions about other contents, such as flavonoid and GABA, are still open for future research. After in vitro digestion, important losses of phenolic compounds are expected. The results obtained are similar to those reported by previous study (Ortega et al., 2011). All phenolic compounds were reduced with percentages of reduction between 15% and 91% compared with the initial concentration.

Many reports showed the potential activities of phenolic compounds in antioxidant, antimicrobial, anti-inflammatory, and analgesic activities (Goyal et al., 2013). The bioaccessibility results for the active compounds suggested that GBR could be considered as a supplement food for heart protection. However, additional experiments should be performed to determine the possible factors responsible for these biological properties of GBR.

## CONCLUSION

This study demonstrates GBR cardioprotective effects on myocardial ischemic hearts. Correlation with these outcomes with antioxidant activity suggests that the effects may be mediated by antioxidant activity of phenolic compounds in GBR. However, definitive proof of cardioprotection mechanism of GBR requires further investigation.
